# Griffipavixanthone, a dimeric xanthone extracted from edible plants, inhibits tumor metastasis and proliferation via downregulation of the RAF pathway in esophageal cancer

**DOI:** 10.18632/oncotarget.6484

**Published:** 2015-12-07

**Authors:** Zhijie Ding, Yuanzhi Lao, Hong Zhang, Wenwei Fu, Lunlun Zhu, Hongsheng Tan, Hongxi Xu

**Affiliations:** ^1^ School of Pharmacy, Shanghai University of Traditional Chinese Medicine, Shanghai, 201203, P.R. China; ^2^ Engineering Research Center of Shanghai Colleges for TCM New Drug Discovery, Shanghai, 201203, P.R. China

**Keywords:** Griffipavixanthone, RAF, esophageal cancer, metastasis, natural product

## Abstract

Metastasis causes a large number of deaths among esophageal cancer patients. The activation of RAF family proteins elevates tumor metastasis and proliferation. In screen targeting the RAF protein, we identified that Griffipavixanthone (GPX), a dimeric xanthone isolated from *Garcinia esculenta*, is a B-RAF and C-RAF inhibitor against esophageal cancer cells. Using wound healing, transwell migration and matrigel invasion assays, we confirmed that GPX significantly inhibited cell migration and invasion. Furthermore, exposure to GPX rendered cell proliferation and induced G2/M cell cycle arrest. Our mechanistic study showed that GPX suppressed cancer metastasis and proliferation through downregulation of RAF-MEK-ERK cascades proteins as well as RAF mRNA levels. In a pulmonary metastasis model, the intraperitoneal injection of GPX significantly suppressed esophageal tumor metastasis and ERK protein level *in vivo*. In conclusion, our present study suggested that GPX could inhibit tumor migration, invasion and proliferation *in vitro* and *in vivo*, which indicated the potential of GPX for preventing and treating esophageal cancer.

## INTRODUCTION

Esophageal adenocarcinoma is the seventh most common tumor that leads to human mortality, and its incidence rate is increasing in China [1–3]. Esophageal adenocarcinoma contains two major forms: squamous cell carcinoma (SCC) and adenocarcinoma, of which over 90% are SCCs [4]. Cancer metastasis is the cause of most patients' death [5], and little progress has been made because of it is a complicated process. For years, great efforts have been taken to treat esophageal cancer [6, 7]; however, due to its aggressiveness and lack of sensitivity to chemotherapy, its treatment remains a challenge.

The RAS-RAF-MAPK pathway is involved in cell proliferation, metastasis and cell survival [8]. High expression of ERK in esophageal cancer leads to a more aggressive phenotype in clinicopathology [9], resulting in tumor malignance. Nevertheless, few studies have been conducted in esophageal SCCs to evaluate this pathway. Recent studies have shown that the first RAF inhibitor approved by the Food and Drug Administration (FDA), Sorafenib (SFB), inhibited cell proliferation that was stimulated by acid or bile acid treatments in esophageal adenocarcinoma by abrogating MAPK activation [10, 11]. Therefore, targeting this cascade seems to be a good choice for treating esophageal cancer metastasis and proliferation [8, 12, 13].

Currently, natural plant-derived compounds are a leading source for drug development [14, 15]. According to our earlier studies, compounds isolated from *Garcinia* species are able to promote apoptosis [16], induce cell cycle arrest [17], and inhibit autophagic flux [18]. Our recent study exhibited the antimetastic effect of oblongifolin C isolated from *Garcinia yunnanensis* Hu by upregulating Keratin 18 and tubulins [19]. All these suggested that compounds from *Garcinia* species contain multiple anticancer activities. In the present study, we performed a screen targeting B-RAF and C-RAF in a high metastatic esophageal adenocarcinoma cell line TE1 using our own library. We identified that Griffipavixanthone (GPX), a dimeric xanthone [20], significantly inhibited the motility and proliferation of esophageal SCC cell lines, suggesting that GPX had a potential application in esophageal cancer cell prevention and therapy.

## RESULTS

### GPX inhibits migration and invasion in TE1 and KYSE150

**Figure 1:**
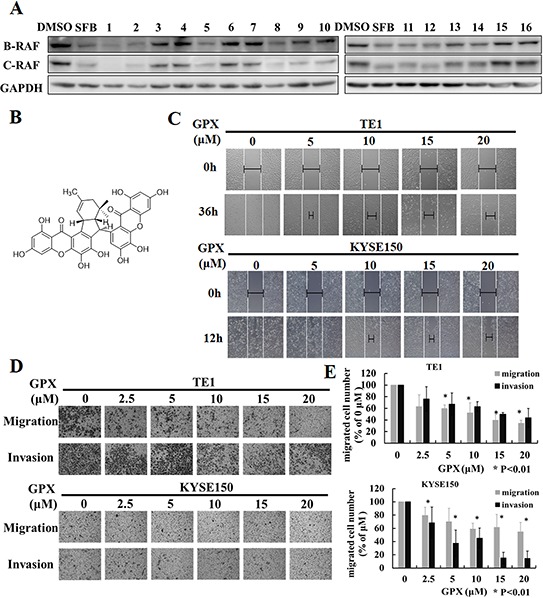
Griffipavixanthone (GPX) inhibits esophageal cancer cell migration and invasion **A.** Screening for B-RAF and C-RAF inhibitors by western blot in esophageal cancer cells. TE1 cell was treated with different compounds (2 μM for compound 1 and 2, 20 μM for other compounds) for 24 h. **B.** Chemical structure of GPX. **C.** Cell migration was examined by wound healing assay. TE1 or KYSE150 were scratched and treated with GPX at concentrations of 0, 5, 10, 15, 20 μM. Images were accessed by a microscope. **D.** Cell migration or invasion was measured by transwell or matrigel coated transwell assays. Cells were incubated with GPX for 24 h, and migrated or invaded cells were fixed and stained with 0.1% crystal violet. **E.** The summary data for transwell migration and invasion assays were presented as the means ± S.D. *P < 0.01.

Because RAF family proteins play essential roles in regulating tumor metastasis and proliferation, we first attempted to screen B-RAF and C-RAF inhibitors from our own *Garcinia* plant compound library using western blotting analysis. The library compounds were obtained from several species of the genus *Garcinia* (Guttiferae) collected from China [21]. As shown in Figure 1A, exposure to some of the compounds in TE1 cells caused the suppression of B-RAF and C-RAF (please refer to Supplementary Table S1 and Supplementary Figure S1 for compounds information). Compounds 1, 2, 5, 8, 12 and 13 exhibited strong inhibition effects compared to the positive control SFB. Among these compounds, compounds 1, 2, 5, 12 and 13 had a strong potential to induce cell death in various cancer cells in our previous study. Interestingly, GPX (compound 8, see Figure 1B for structure) reduced the expression of B-RAF and C-RAF with minor cytotoxicity in several esophageal cancer cells (Supplementary Figure S2). We then chose GPX to further study its effect on cell migration and invasion. In the wound healing assay, GPX inhibited TE1 and KYSE150 cell migration in a dose dependent manner (Figure 1C). Furthermore, transwell migration and the matrigel invasion assay indicated that GPX efficiently suppressed cell migration and invasion (Figure 1D). The inhibition of GPX at 10 μM was 48 ± 17% on TE1 for migration and 47 ± 9% for invasion (Figure 1E, upper panel). Similarly, migrated cells of KYSE150 decreased by 42 ± 9% and invaded cells decreased by 55 ± 15% when treated with GPX at 10 μM (Figure 1E, lower panel). These data suggest that GPX suppressed the expression of B-RAF and C-RAF and esophageal cell metastasis *in vitro*.

### GPX inhibits colony formation and induces cell cycle arrest

**Figure 2:**
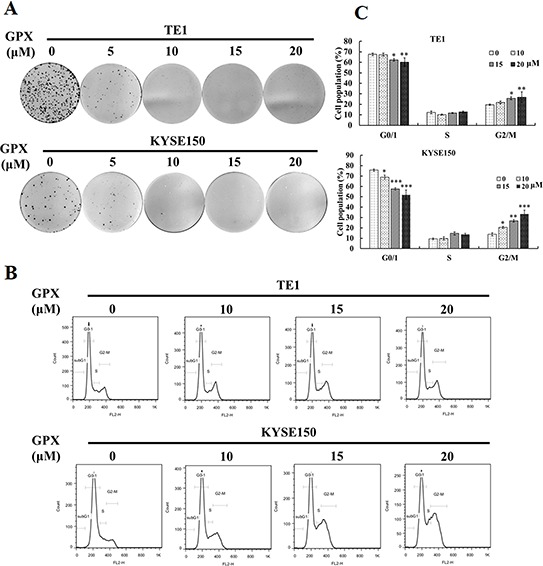
GPX inhibits cell proliferation and induces G2/M arrest in TE1 and KYSE150 cells **A.** Colony formation assay. TE1 or KYSE150 cells were colonized for 7 days after incubation with GPX with indicated doses for 48 h. **B.** Cell cycle distribution under GPC treatment. Cells were treated with indicated doses of GPX (0, 10, 15, 20 μM) for 48 h, fixed, stained with PI, and analyzed by flow cytometry. **C.** Graphs for the percentage of cell cycle distribution are displayed. Data are presented as the means ± S.D. *P < 0.05, **P < 0.01, ***P < 0.001.

To investigate the effect of GPX on cell proliferation, a colony formation assay was applied. As shown in Figure 2A, TE1 and KYSE150 cells treated with 10 μM GPX for 48 h could not form colonies, indicating that GPX inhibits the proliferation of these cell lines. We then further explored the effects of GPX on the cell cycle distribution. TE1 and KYSE150 cells were incubated with various concentrations of GPX for 48 h and were analyzed by flow cytometry with DNA staining. Dose-dependent G2/M arrest was observed (Figure 2B), and the statistical analysis showed that GPX caused G2/M cell cycle arrest in TE1 and KYSE150 cells (Figure 2C). Additionally, we examined the effect on another esophageal cell Eca109. As shown in Supplementary Figure S2A and S2B, GPX induced G0/1 arrest on Eca109 within 48 h. To access the effects on GPX on cell death, we applied MTT assay, SYBRE-Green assay, morphology observation, and PI/Annexin-V double staining. As shown in Supplementary Figure S3 and S4, GPX did not cause cell death in all the tested conditions. In short conclusion, GPX exhibits tumor suppression effects on metastasis and proliferation without obvious cytotoxicity *in vitro*.

### GPX prevents pulmonary metastasis in a mouse model

**Figure 3:**
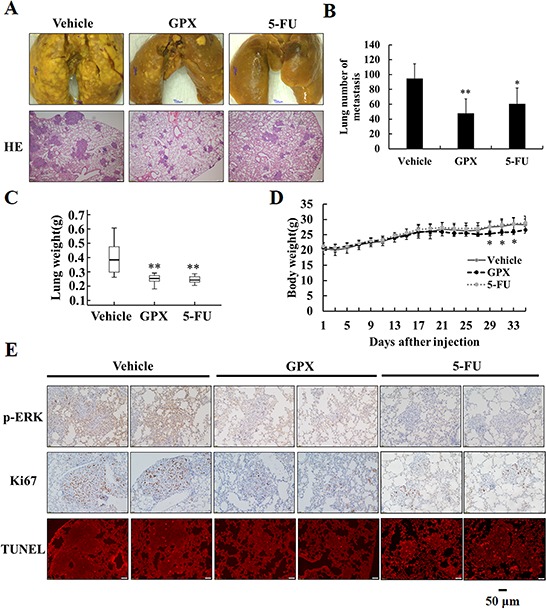
GPX prevents esophageal pulmonary metastasis *in vivo* Six-week-old male nude mice were tail vein injected with 1 × 10^6^ KYSE150 cells. After injection, the mice were divided into three groups, administered DMSO (vehicle group), GPX (20 mg/kg per 2 days) or 5-FU (20 mg/kg per 2 days) via intraperitoneal injection (*n* = 8 in each group). **A.** Representative examples and HE staining of lungs from each group after 5 weeks. **B.** Quantitative analysis of metastatic nodes in lung. **C.** Lung weight analysis after treatment. **D.** Body weight analysis every two days during the whole expermients. **E.** Immunohistochemistry staining of p-ERK, Ki-67 and TUNEL staining in lung tissues. Data are presented as the means ± S.D. *P < 0.05, **P < 0.01.

We then explored the metastatic inhibition effect of GPX *in vivo* using the tail vein injection pulmonary metastasis mouse model. After KYSE150 cells injection, the mice were randomly divided into three groups and administered DMSO, GPX or 5-FU via intraperitoneal injection (*n* = 8 in each group). Thirty-five days after tumor injection, the mice were sacrificed, and the pulmonary metastasis was examined by HE and immunohistochemistry staining. As shown in the upper panel of Figure 3A, lung tumor nodules were observed in the control group, whereas both GPX and 5-FU reduced the tumor nodules. As shown in the lower panel, HE staining showed the large size of the metastatic foci in the control group and showed that the foci were sparse and smaller in mice treated with GPX and 5-FU, which was confirmed by the statistical analysis in Figure 3B. Additionally, the weight of the lungs in the GPX and 5-FU treated groups decreased significantly compared to that in the control group (Figure 3C). Consistent with the *in vitro* experiments, GPX did not cause significant side effects to the mice because only a minor reduction in weight loss was observed (Figure 3D). To investigate the effects of GPX on cell cycle and cell death, we performed immunohistochemistry to detect phospho-ERK, Ki67 and TUNEL staining in the lung tissues. As shown in Figure 3E, phospho-ERK and Ki67 were remarkably suppressed in both the GPX and 5-FU treated groups. Other tissues including liver, kidney, heart, and spleen did not show obvious morphological changes (Supplementary Figure S5A). In addition, the pathological analysis indicated that there was no liver metastasis as shown in Supplementary Figure S5B. In summary, our *in vivo* study indicated that GPX suppressed pulmonary metastasis without significant side effects against other organs in nude mice.

### GPX attenuates RAF-MAPK signaling pathway

**Figure 4:**
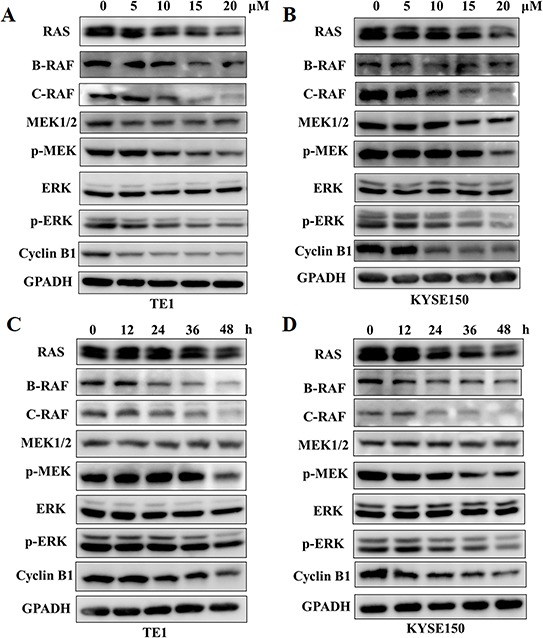
GPX downregulates RAS-RAF-MEK-ERK cascades and cyclinB1 protein levels RAS-RAF-MEK-ERK cascades and cyclinB1 proteins were analyzed by western blot. **A.** and **B.** TE1 or KYSE150 cells were treated with GPX with various concentrations of GPX (0, 10, 15, 20 μM) for 48 h. **C.** and **D.** TE1 or KYSE150 cells were treated with GPX at a dose of 20 μM for different time points.

**Figure 5:**
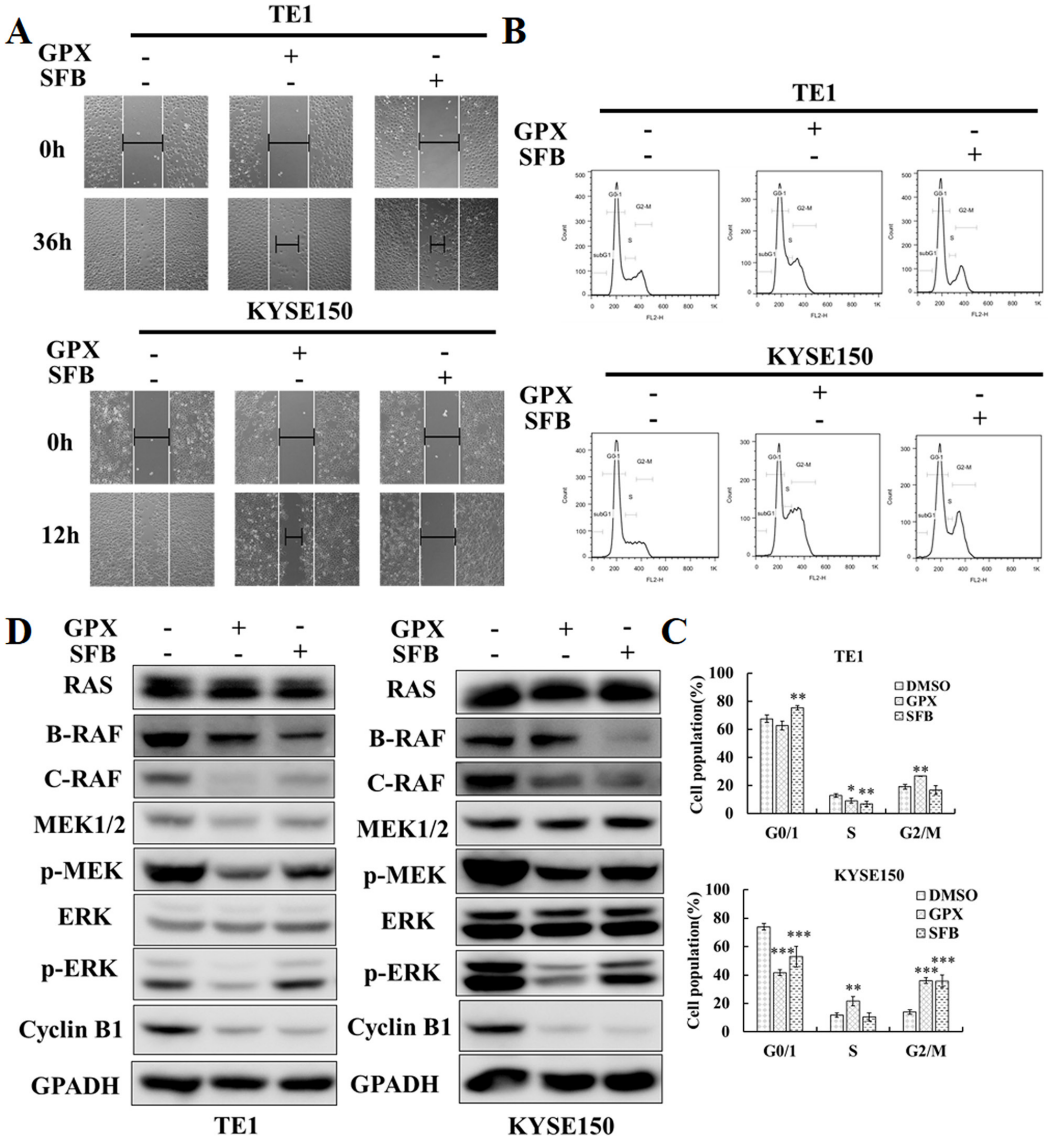
GPX exhibits similar effects on cell migration, cell cycle, and RAS-RAF-MEK-ERK cascades compared with Sorafenib **A.** Would healing assay. Cells were treated with 20 μM GPX or Sorafenib. **B.** Cell cycle distribution. TE1 and KYSE150 cells were treated with 20 μM GPX or SFB for 48 h. **C.** Statistic analysis on cell cycle distribution. Data are presented as the means ± S.D. *P < 0.05, **P < 0.01, ***P < 0.001. **D.** RAS-RAF-MAPK cascade related proteins and cyclin B1 were analyzed by western blot. TE1 and KYSE150 cells were treated with 20 μM GPX or SFB for 48 h.

To confirm the effects of GPX on B-RAF and C-RAF proteins, we systematically examined the RAS-RAF-MAPK cascades in TE1 and KYSE150 cells. As shown in Figure 4A and 4B, GPX decreased the B-RAF, C-RAF, phospho-MEK and phospho-ERK protein levels in a dose-dependent manner, without significantly changing the protein level of total RAS, MEK and ERK. A similar effect was also observed in Eca109 cells (Supplementary Figure S2C). Similarly, the B-RAF, C-RAF and phospho-MAPK protein levels were attenuated in a time dependent manner after treatment with 20 μM GPX (Figure 4C and 4D). We also examined whether GPX influenced G2/M arrest through the cyclinB1 protein. As shown in Figure 4, GPX decreased the cyclinB1 protein levels in cancer cells in a dose and time dependent manner. In TE1 and KYSE150, 5 μM and 10 μM of GPX, respectively, decreased the protein levels. The effective times were 24 h and 12 h, respectively. We further investigated the mRNA levels of B-RAF and C-RAF after GPX treatment. Interestingly, these two mRNAs were downregulated in both cell lines (Supplementary Figure S6), indicating that GPX also affected B-RAF and C-RAF at the transcriptional level. Furthermore, we carefully compared the effects of GPX on migration, cell cycle distribution, and the RAF-MAPK cascade with Sorafenib, a commercial RAF inhibitor. As shown in Figure. 5A, treatment with GPX and Sorafenib at a concentration of 20 μM could suppress TE1 and KYSE150 cell migration. GPX and Sorafenib induced a similar cell cycle arrest in TE1 and KYSE150 cells (Figure 5B and 5C). Western blotting analysis showed that B-RAF, C-RAF, phosphor-MEK, phosphor-ERK and cyclinB1 were significantly decreased, whereas RAS was not affected by GPX or Sorafenib (Figure 5D). Taken together, these results suggest that GPX inhibits the tumor metastatic capability and induces cell cycle arrest by attenuating RAF-MAPK signaling pathways.

### GPX inhibits AKT and epithelial-mesenchymal transition signaling pathways

**Figure 6:**
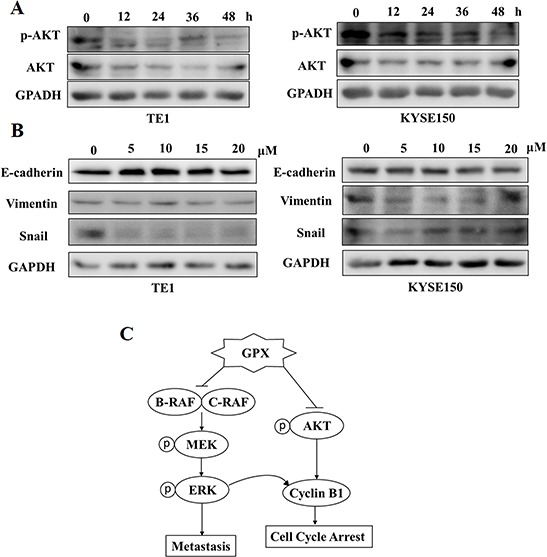
GPX inhibits AKT phosphorylation and epithelial mesenchymal transition (EMT) proteins **A.** AKT phosphorylation was examined by western blot. TE1 and KYSE150 cells were treated with 20 uM GPX for indicated time periods. **B.** Three EMT markers: E-cadherin, Vimentin and Snail were measured by western blot. TE1 and KYSE150 cells were treated with 20 uM GPX for indicated time periods. **C.** The hypothesized mechanism of action of GPX in esophageal cancer cells.

AKT is an EGFR downstream signal and is involved in cell proliferation, angiogenesis, and metastasis in head and neck cancer [11]. We then checked the effect of GPX on the phosphorylation of AKT in TE1 and KYSE150 cells. As shown in Figure 6A and Supplementary Figure S7, GPX caused a decrease in the phosphorylation of AKT in both dose and time dependent manners. Matrix metalloproteinases (MMPs), including MMP-2 and MMP-9, play essential roles in regulating tumor metastasis and angiogenesis [22]. We performed quantitative RT-PCR to investigate the effect of GPX on MMP-2 and MMP-9 mRNA levels. As shown in Supplementary Figure S8, GPX caused the decrease in MMP-2 mRNA in a time-dependent manner, whereas the MMP-9 mRNA was not altered significantly.

The epithelial-mesenchymal transition (EMT) is a fundamental process in embryogenesis, functioning as a main character in cancer progression, including endowing cells with migratory and invasive properties [23]. E-cadherin, Vimentin, and Snail are three EMT markers that regulate cell motility; Snail is a C2H2 zinc finger protein that promotes EMT, whereas E-cadherin is a cell adhesion molecule and Vimentin is a mesenchymal marker [24]. We are curious how GPX regulates these three proteins. Interestingly, GPX exhibited various effects on TE1 and KYSE150 cells. As shown in Figure 6B and Supplementary Figure S9, GPX mainly decreased Snail expression in TE1 cells and Vimentin experssion in KYSE150 respectively. Nevertheless, GPX showed a profound impact on multiple signaling pathways, including metastasis, cell cycle arrest, and EMT.

In summary, our study indicated that GPX was an interesting natural compound affecting multiple signals in esophageal cancer *in vitro* and *in vivo*, as summarized in Figure 6C. Our data suggested that GPX may be a promising candidate to be developed as an anticancer compound in cancer prevention and therapy.

## DISCUSSION

The RAF-MEK-ERK pathway is an important drug target in cancer therapy [13]. However, there have been limited studies that have searched for RAF kinase inhibitors from natural compounds. In the present study, we tried to screen novel B-RAF and C-RAF inhibitors from natural compounds using cell based western blotting assays. We found that GPX was an efficient RAF inhibitor without significant cytotoxicity. We then demonstrated the anti-metastasis and anti-proliferation effects of GPX on esophageal cancer *in vitro* and *in vivo*. Our further studies indicated that GPX might be an effective inhibitor of tumor metastasis, which was investigated by using wound healing, transwell migration and invasion assays. Simultaneously, we showed that GPX could decrease cell colonies and trigger cell cycle arrest at the G2/M phase. We also demonstrated that the mechanisms of action of GPX included the inhibition of RAF-MAPK signaling pathways, the decrease of cyclinB1, and the downregulation of the EMT pathway.

Xanthones, benzophenone, and polycyclic polyprenylated acylphloroglucinols (PPAPs) are the major chemicals from *Garcinia* species plants containing multiple bioactivities, including an anticancer effect [25, 26]. We were curious whether the fruits contained any effective compounds with anticancer potential. In this study, we found a dimeric xanthone named GPX to be an active metastatic inhibitor in esophageal cancers. Our results indicated that GPX had a similar effect to a commercial RAF inhibitor, Sorafinib (Figure 5). However, *in silico* docking simulation suggested that GPX might not directly bind to B-RAF or C-RAF due to its large molecular weight (data not shown). Our data also suggested that GPX inhibited the RAF pathway at least partially through the downregulation of B-RAF and C-RAF mRNA levels (Supplementary Figure S3). It will be interesting to further investigate whether GPX can regulate RAF through other mechanisms, such as protein stabilities. Additionally, the detailed mechanisms on how GPX affects cyclinB1 and EMT pathways also need to be further explored.

GPX showed lower cytotoxicity than other compounds, such as caged xanthones (e.g., Gambogic acid) and PPAPs (e.g., Oblongifolin C, Guttiferone K), in our preliminary study (data not shown). In our animal study, TUNEL staining indicated that GPX did not cause significant cell death in the xenograft tissue compared to the positive control 5-FU (Figure 3E). Because GPX is extracted from edible fruits, the data implicate that this fruit might have the potential to be developed as a functional food for cancer prevention or therapy. An epidemiological study indicated that the incidence rates of esophageal cancer are related to several factors, such as cigarette smoking, alcohol drinking, and dietary habits [3]. It is still unclear if the uptake of the fruits is correlated to the occurrence of esophageal cancer from the literature. To elucidate whether the long term uptake of the fruits of *Garcinia esculenta* can benefit cancer prevention, more detailed studies in animal models are required.

## MATERIALS AND METHODS

### Chemicals and reagents

Griffipavixanthone was isolated from twigs of *Garcinia esculenta* as previously described [27]. Phosphate buffered saline (PBS), 3-(4,5-dimethylthiazol-2-yl)-2,5-diphenyltetrazoliumbromide (MTT), Propidium iodide (PI), Bouin's solution and 5-FU were purchased from Sigma-Aldrich (St. Louis, MO, USA). Matrigel was purchased from BD (San Jose, CA, USA). Sorafenib Tosylate was purchased from Selleck Chemicals (Houston, TX, USA).

### Cell culture

The human esophageal cancer cell line TE1 (obtained 2013, STR tested May 2015) and the KYSE150 cells (obtained 2013, STR tested May 2015) were provided from Fudan University Shanghai Cancer Center. Cells were grown in RPMI 1640 medium (Hyclone, Logan, UT, USA) supplemented with 10% fetal bovine serum (FBS), 100 U/ml penicillin and 100 mg/ml streptomycin in a humidified atmosphere containing 5% CO_2_ at 37°C.

### Cell viability assay

An MTT assay was used to assess cell viability. Cells (1 × 10^4^) were seeded into a 96-well culture plate for 24 h. Cells were incubated with or without serial dilutions of GPX. After 24 and 48 h, 10 μl of MTT (5 mg/ml) was added and incubated for 4 h. Dimethyl sulfoxide was used to dissolve the formazan crystals, and the absorbance at 570 nm was measured by a microplate reader.

### Wound healing assay

Wound healing was used to evaluate cell motility. Cells (1 × 10^5^) were seeded into a 24-well culture plate. When the cells grew to 80–90% confluence, a scratch was created through the cell monolayer by sterile 100 μl pipette tips, and fresh medium with or without GPX was added. The cell migration was observed and imaged under an IX83 microscope (Olympus, Tokyo, Japan).

### Transwell migration and invasion assay

Cell migration and invasion were estimated using transwell chambers (Corning, NY, USA) with a pore size of 8 μm. For the migration assay, a total of 5 × 10^4^ cells were added into the upper chamber in serum-free medium, and in the bottom chamber, 600 μL of 10% FBS medium was added. After incubating with various concentrations of GPX for 24 h, the cells on the upper surface of the chamber were removed using cotton swabs, and then, the migrated cells on the bottom surface were fixed in 4% paraformaldehyde, stained with 0.1% crystal violet and scored under a light microscope in five random fields. As for the transwell invasion assay, the upper chamber membranes were pre-incubated with matrigel (BD Biosciences, Bedford, MA, USA) for 2 hours at 37°C.

### Colony formation assay

For the colony formation assay, cells (500 per/well for TE1 and 250 per/well for KYSE150) were seeded in a 6-well culture plate and cultured for 7 days after incubation with GPX for 48 h. The colonies were fixed in 4% paraformaldehyde, stained with 0.1% crystal violet and imaged.

### Flow cytometry

Cells (2 × 10^5^) were plated in a 6-well culture plate and maintained with GPX for 48 h. Then, the cells were harvested, fixed in 70% cold ethanol and stored at 4°C overnight. The cells were incubated with PI containing RNaseA for 30 min and analyzed by Flow Cytometry (BD Biosciences Inc., Franklin Lakes, NJ, USA).

For FITC Annexin V and PI staining, cells were harvested, suspended in 500 μl binding buffer and incubated with 5 μl FITC Annexin V and 10 μl PI for 15 min. Then, the mixtures were detected under Flow Cytometry.

### Quantitative real-time PCR

Total RNA was extracted from GPX treated TE1 or KYSE150 cells using Trizol (Takara, Shiga, Japan) according to the manufacturer's directions. Then, RNA was reverse transcribed with the use of a PrimeScript RT reagent kit. Quantitative PCR was conducted with forward and reverse primers containing SYBER Green. Then, real-time PCR was performed under a StepOnePlus Real-Time PCR System. The primers for human genes were as follows: for MMP-2, forward primer: 5′-GTGCTGAAGGACACACTAA-3′, reverse primer: 5′-TTGCGAGGGAAGAAGTTG-3′, for MMP-9, forward primer: 5′-TTTGACAGCGACAAGAAGT-3′, reverse primer: 5′-CTCAGTGAAGCGGTACATA-3′, for GAPDH, forward primer: 5′-TGTTGCCATCAATGACCCCTT-3′, reverse primer: 5′-CTCCACGACGTACTCAGCG-3′, for B-RAF, forward primer: 5′-CATTGGTTTTGATGAGTATATGAAC-3′, reverse primer: 5′-GGAGACACTTTGTAGCAGAG-3′ and for C-RAF, forward primer: 5′- TGAGCACTGTAGCACCAAAGTACCT-3′, reverse primer: 5′-CAGACTCTCGCATACGACGCAT-3′.

### Western blot analysis

TE1 and KYSE150 cells were seeded on a 3.5-cm dish, treated with GPX at various concentrations and times, and lysed in RIPA buffer. Proteins were separated on SDS polyacrylamide gels and transferred to PVDF membranes (Millipore, Billerica, MA, USA). The membranes were blocked and immunoblotted with primary antibodies at 4°C overnight, followed by appropriate secondary antibodies. GAPDH was used as the loading control. Membranes were visualized under Image Quant LAS 4000 mini and processed by Image Quant TL 1D software (General Electric Company).

For transfected cells, 3 × 10^5^ KYSE150 cells were seeded in 6-well plate. After 24 h, they were transfected with 2 μg plasmid and changed medium 6 h later. p-ERK and ERK protein level were detected 24 h and 48 h later.

The primary antibodies C-RAF (Cat.9422), MEK1/2 (Cat.9122), p-MEK (Ser217/221, Cat.9154), ERK (Cat.4695), p-ERK (Tyr202/Tyr204, Cat.4370), GAPDH (Cat.5174), p-AKT (Ser473, Cat.9171), cyclinB1 (Cat.12231), E-cadherin (Cat.3195), vimentin (Cat.5741), and snail (Cat.3879) were purchased from Cell Signaling Technologies (Danvers, MA, USA). RAS (Cat.ab137739) and B-RAF (Cat.ab33899) were purchased from Abcam (Cambridge, UK).

### Animal study, immunohistochemistry and TUNEL assay

The *in vivo* study was performed as described previously [28]. Briefly, 1 × 10^6^ KYSE150 cells were intravenously injected into six-week-old male nude mice (Experimental Animal Center of the Chinese Academy of Science, Shanghai, China). After injection of esophageal cancer cells, the mice were divided into 3 groups randomly (*n* = 8 in each group) and DMSO, GPX (20 mg/kg) or 5-FU (20 mg/kg) were administered via an intraperitoneal injection every other day. Body weight was measured every 2 days. At 35 days, the lungs were removed and fixed in Bouin's solution. The numbers of lung nodules were counted and confirmed by HE staining. The immunohistochemistry of p-ERK and Ki-67 were performed in the lungs. A TUNEL assay was performed to detect the fragmented DNA of the apoptotic pulmonary nodules. After being extracted and fixed, the sections of lungs were stained by the terminal deoxynucleotidyl transferase-mediated dUTP-biotin nick end labeling (TUNEL) method using an in situ cell death detection kit (Roche, Penzberg, Germany) and imaged under a fluorescence microscope.

### SYBR green assay

For SYBR green assay, cells (1 × 10^4^) were seeded into a 96-well culture plate for 24 h. Then, they were incubated with or without various dilutions of GPX for 24 and 48 h. After that, the medium was removed, and the cells were incubated with 100 μ l of SYRB green (1:10,000) in lysis buffer. 30 min later, the absorbance of the fluorescence was read under a microplate Reader.

### DAPI staining

For DAPI staining, TE1 and KYSE150 cells (1 × 10^5^) were plated on a 3.5-cm dish. After 24 h, cells were treated with 20 μM GPX for different times (24 h and 48 h). Then, they were fixed in 4% formaldehyde, permeabilized in 0.3% Triton X-100 and stained in DAPI. The cell apoptosis was observed and imaged under an IX83 microscope (Olympus, Tokyo, Japan).

### Statistical analysis

All of the results were repeated at least three times, and data were analyzed by SPSS 18.0 software and described as the means ± S.D. Statistically significant differences between two independent groups were determined by two-tailed Student's *t*-test, and 3 or more group comparisons were evaluated by one-way ANOVA. A value of *P* < 0.05 was defined as significant.

## SUPPLEMENTARY FIGURES AND TABLE



## References

[1] Siegel R, Ma J, Zou Z, Jemal A (2014). Cancer statistics, 2014. CA: a cancer journal for clinicians.

[2] Simard EP, Ward EM, Siegel R, Jemal A (2012). Cancers with increasing incidence trends in the United States: 1999 through 2008. CA: a cancer journal for clinicians.

[3] Lin Y, Totsuka Y, He Y, Kikuchi S, Qiao Y, Ueda J, Wei W, Inoue M, Tanaka H (2013). Epidemiology of Esophageal Cancer in Japan and China. Journal of Epidemiology.

[4] Stoner GD, Gupta A (2001). Etiology and chemoprevention of esophageal squamous cell carcinoma. Carcinogenesis.

[5] Steeg PS (2006). Tumor metastasis: mechanistic insights and clinical challenges. Nat Med.

[6] Gaur P, Kim MP, Dunkin BJ (2014). Esophageal cancer: Recent advances in screening, targeted therapy, and management. Journal of carcinogenesis.

[7] Ku GY, Ilson DH (2013). Emerging tyrosine kinase inhibitors for esophageal cancer. Expert opinion on emerging drugs.

[8] Roberts PJ, Der CJ (2007). Targeting the Raf-MEK-ERK mitogen-activated protein kinase cascade for the treatment of cancer. Oncogene.

[9] Tasioudi KE, Saetta AA, Sakellariou S, Levidou G, Michalopoulos NV, Theodorou D, Patsouris E, Korkolopoulou P (2012). pERK activation in esophageal carcinomas: clinicopathological associations. Pathology, research and practice.

[10] Delgado JS, Mustafi R, Yee J, Cerda S, Chumsangsri A, Dougherty U, Lichtenstein L, Fichera A, Bissonnette M (2008). Sorafenib triggers antiproliferative and pro-apoptotic signals in human esophageal adenocarcinoma cells. Digestive diseases and sciences.

[11] Keswani RN, Chumsangsri A, Mustafi R, Delgado J, Cohen EE, Bissonnette M (2008). Sorafenib inhibits MAPK-mediated proliferation in a Barrett's esophageal adenocarcinoma cell line. Diseases of the esophagus.

[12] Sebolt-Leopold JS, Herrera R (2004). Targeting the mitogen-activated protein kinase cascade to treat cancer. Nature reviews Cancer.

[13] Montagut C, Settleman J (2009). Targeting the RAF-MEK-ERK pathway in cancer therapy. Cancer letters.

[14] Unnati S, Ripal S, Sanjeev A, Niyati A (2013). Novel anticancer agents from plant sources. Chin J Nat Medicines.

[15] Newman DJ, Cragg GM (2012). Natural Products As Sources of New Drugs over the 30 Years from 1981 to 2010. Journal of natural products.

[16] Feng C, Zhou LY, Yu T, Xu G, Tian HL, Xu JJ, Xu HX, Luo KQ (2012). A new anticancer compound, oblongifolin C, inhibits tumor growth and promotes apoptosis in HeLa cells through Bax activation. International journal of cancer.

[17] Kan WL, Yin C, Xu HX, Xu G, To KK, Cho CH, Rudd JA, Lin G (2013). Antitumor effects of novel compound, guttiferone K, on colon cancer by p21Waf1/Cip1-mediated G(0) /G(1) cell cycle arrest and apoptosis. International journal of cancer.

[18] Lao Y, Wan G, Liu Z, Wang X, Ruan P, Xu W, Xu D, Xie W, Zhang Y, Xu H, Xu N (2014). The natural compound oblongifolin C inhibits autophagic flux and enhances antitumor efficacy of nutrient deprivation. Autophagy.

[19] Wang X, Lao Y, Xu N, Xi Z, Wu M, Wang H, Li X, Tan H, Sun M, Xu H (2015). Oblongifolin C inhibits metastasis by up-regulating keratin 18 and tubulins. Scientific reports.

[20] Merza J, Aumond MC, Rondeau D, Dumontet V, Le Ray AM, Seraphin D, Richomme P (2004). Prenylated xanthones and tocotrienols from Garcinia virgata. Phytochemistry.

[21] Xu L, Lao Y, Zhao Y, Qin J, Fu W, Zhang Y, Xu H (2015). Screening Active Compounds from Garcinia Species Native to China Reveals Novel Compounds Targeting the STAT/JAK Signaling Pathway. BioMed research international.

[22] Curran S, Murray GI (2000). Matrix metalloproteinases: molecular aspects of their roles in tumour invasion and metastasis. European journal of cancer.

[23] Thiery JP, Acloque H, Huang RY, Nieto MA (2009). Epithelial-mesenchymal transitions in development and disease. Cell.

[24] Smith BN, Burton LJ, Henderson V, Randle DD, Morton DJ, Smith BA, Taliaferro-Smith L, Nagappan P, Yates C, Zayzafoon M, Chung LW, Odero-Marah VA (2014). Snail promotes epithelial mesenchymal transition in breast cancer cells in part via activation of nuclear ERK2. PloS one.

[25] Han QB, Wang YL, Yang L, Tso TF, Qiao CF, Song JZ, Xu LJ, Chen SL, Yang DJ, Xu HX (2006). Cytotoxic polyprenylated xanthones from the resin of Garcinia hanburyi. Chem Pharm Bull (Tokyo).

[26] Richard JA, Pouwer RH, Chen DY (2012). The chemistry of the polycyclic polyprenylated acylphloroglucinols. Angew Chem Int Ed Engl.

[27] Zhang H, Zhang DD, Lao YZ, Fu WW, Liang S, Yuan QH, Yang L, Xu HX (2014). Cytotoxic and anti-inflammatory prenylated benzoylphloroglucinols and xanthones from the twigs of Garcinia esculenta. Journal of natural products.

[28] Chen RS, Song YM, Zhou ZY, Tong T, Li Y, Fu M, Guo XL, Dong LJ, He X, Qiao HX, Zhan QM, Li W (2009). Disruption of xCT inhibits cancer cell metastasis via the caveolin-1/beta-catenin pathway. Oncogene.

