# Supplementary material for: Griffipavixanthone, a dimeric xanthone extracted from edible plants, inhibits tumor metastasis and proliferation via downregulation of the RAF pathway in esophageal cancer
Source: Oncotarget. 2015 Dec 7;7(2):1826–37. doi: 10.18632/oncotarget.6484 (PMC4811500; doi:10.18632/oncotarget.6484)
Supplement: Supplementary file 1 [file oncotarget-07-1826-s001.pdf]

SUPPLEMENTARY FIGURES AND TABLE

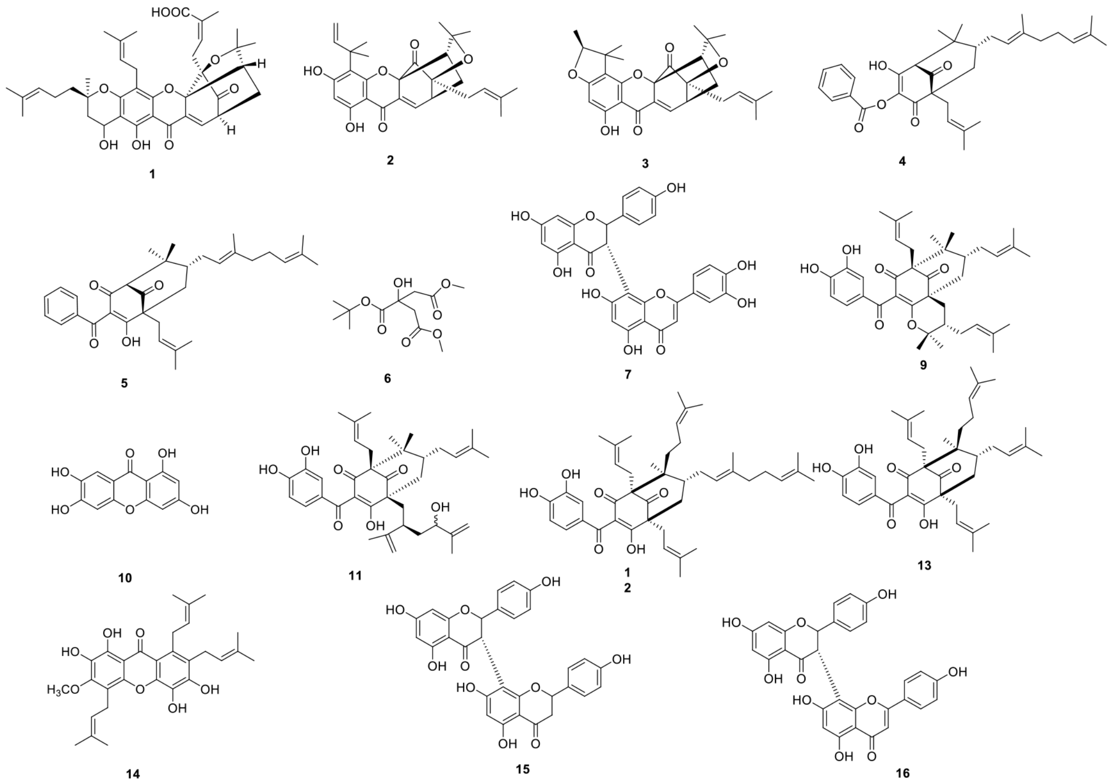

Supplementary Figure S1: Chemical structure of compounds in Supplementary Table S1.

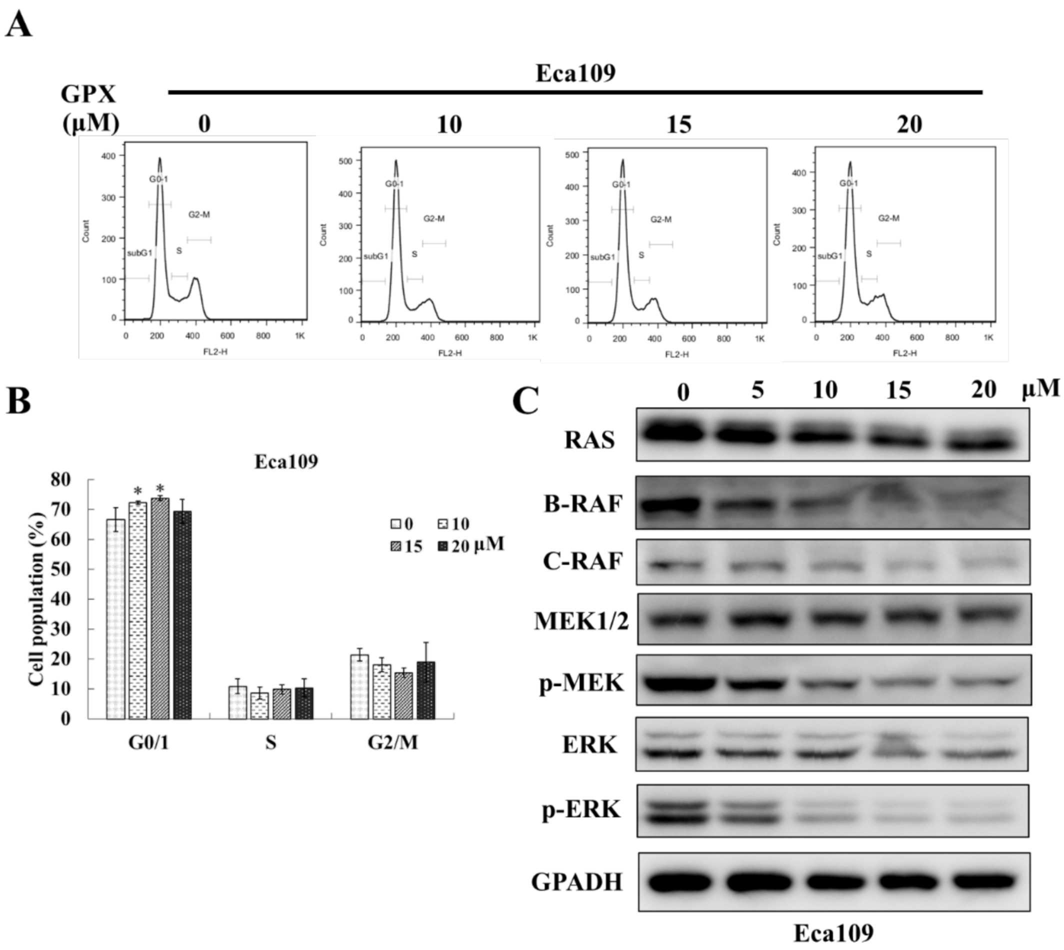

Supplementary Figure S2: GPX induces G0/1 arrest and downregulates RAS-RAF-MEK-ERK cascades in Eca109 cells. A. Cell cycle distribution under GPX treatment. Cells were treated with DMSO and various concentrations of GPX (10, 15, 20 μM) for 48 h, fixed, labeled and analyzed by flow cytometry. **B.** Statistic analysis of cell cycle distribution from (A) Data are presented as the means ± S.D. $*P < 0.05$. **C.** Western blot of RAS-RAF-MEK-ERK cascade proteins under GPX treatment in Eca109 cells. Cells were treated with GPX for 48 h, protein lysates were prepared and proteins were analyzed by western blot.

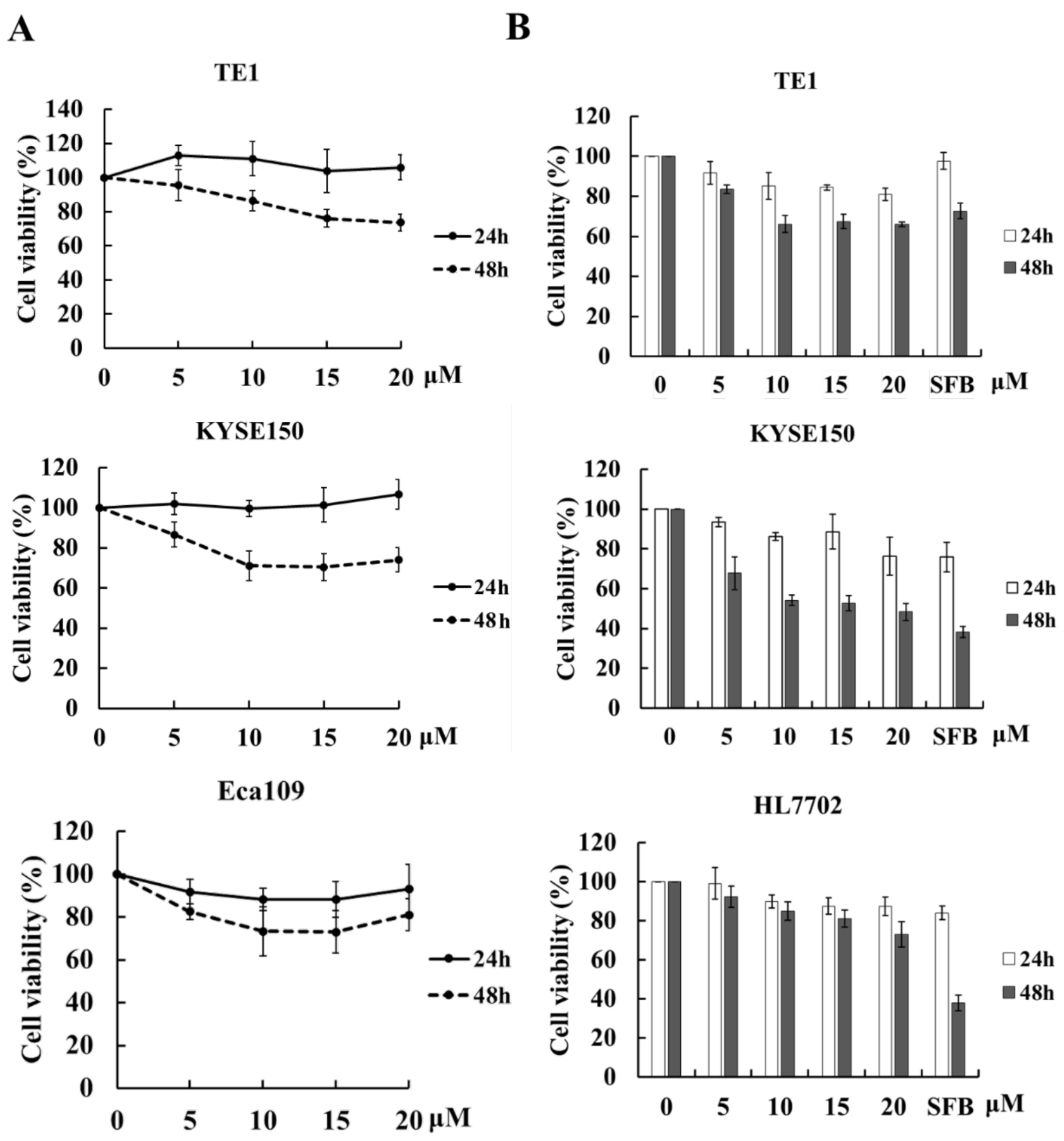

Supplementary Figure S3: Effects on cell viability of GPX on esophageal cancer cells. A. TE1, KYSE150 and Eca109 cell proliferations were accessed by MTT assay. Cells were treated with various concentrations of GPX, added to MTT, dissolved by DMSO and measured at 570 nm. **B.** TE1, KYSE150 and HL7702 cell proliferations were accessed by Syber green assay. 20 µM SFB was used as positive control.

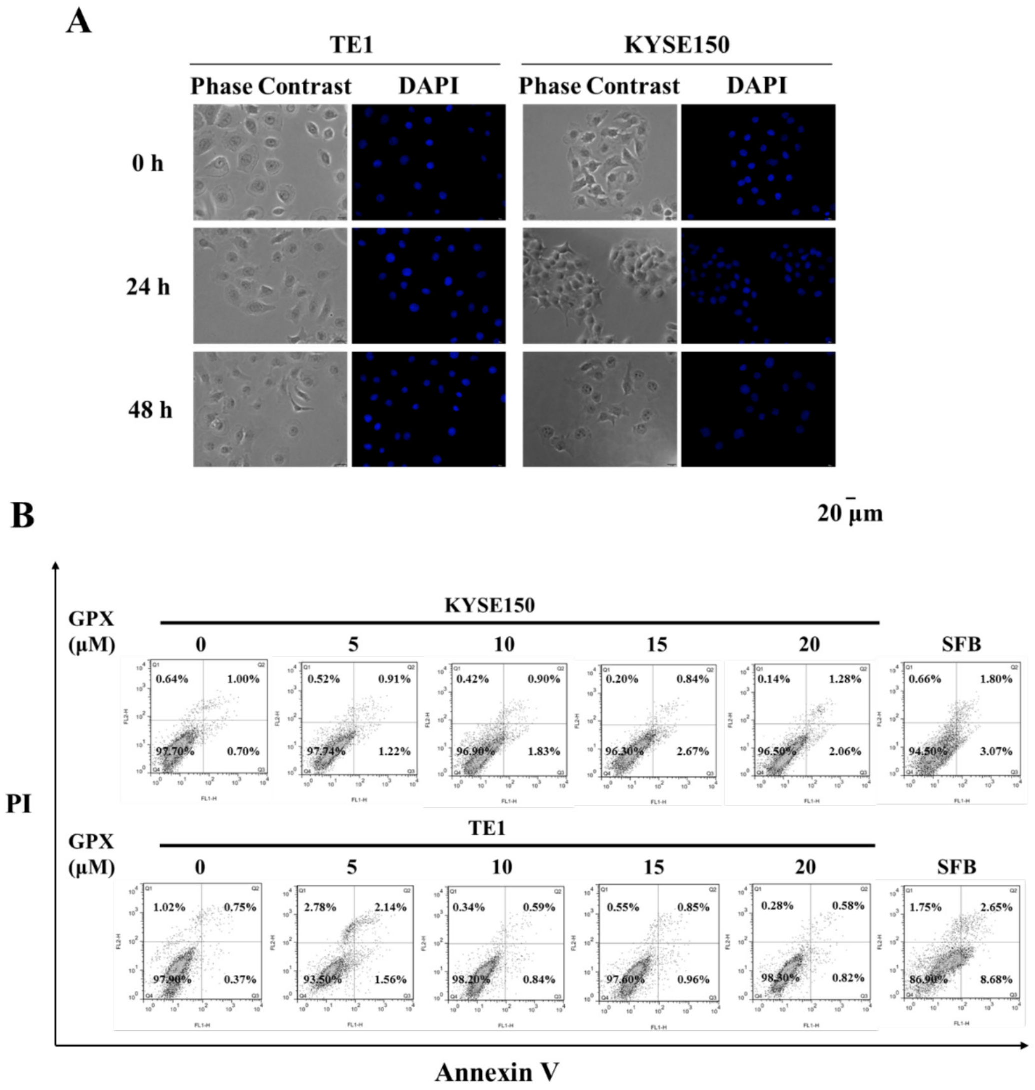

Supplementary Figure S4: GPX does not cause apoptosis in esophageal cancer cells. A. Cell morphology and DNA content under GPX treatment. TE1 and KYSE150 cells (1×10^5) were plated on a 3.5-cm dish. After 24 h, cells were treated with 20 µM GPX for different times (24 h and 48 h). Then, they were fixed and stained in DAPI. Cell images were acquired by Olympus fluorescent Microscope. **B.** Annexin V/PI double staining under GPX treatment. Cells were seeded and exposed to various concentrations of GPX (0, 5, 10, 15, 20 µM). Then, they were harvested and suspended in 500 µl binding buffer, incubated with 5 µl FITC Annexin V and 10 µl PI for 15 min, and analyzed by flow cytometry. 20 µM SFB was used as positive control.

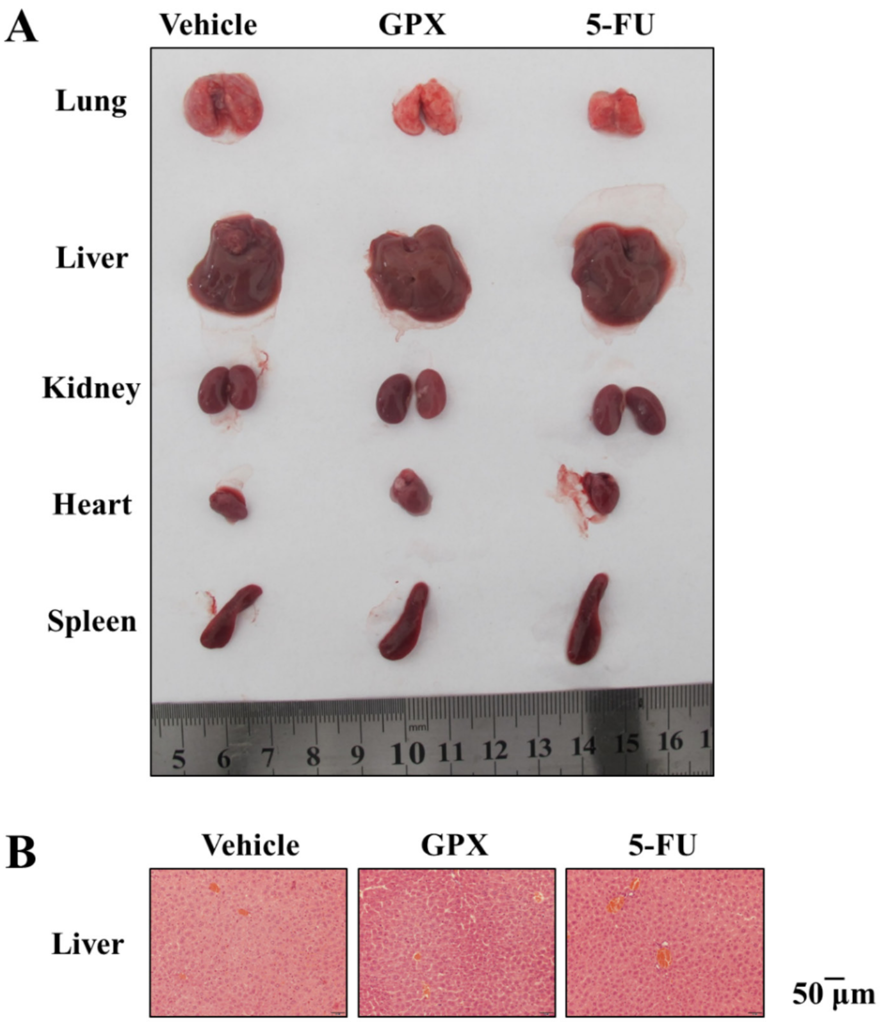

Supplementary Figure S5: GPX shows low toxicity *in vivo*. Six-week-old male nude mice were treated with GPX for 35 days. **A.** Images of lung, liver, kidney, heart and spleen from vehicle, GPX (20mg/kg) and 5-FU (20mg/kg) treated nude mice. **B.** HE staining of liver was exhibited.

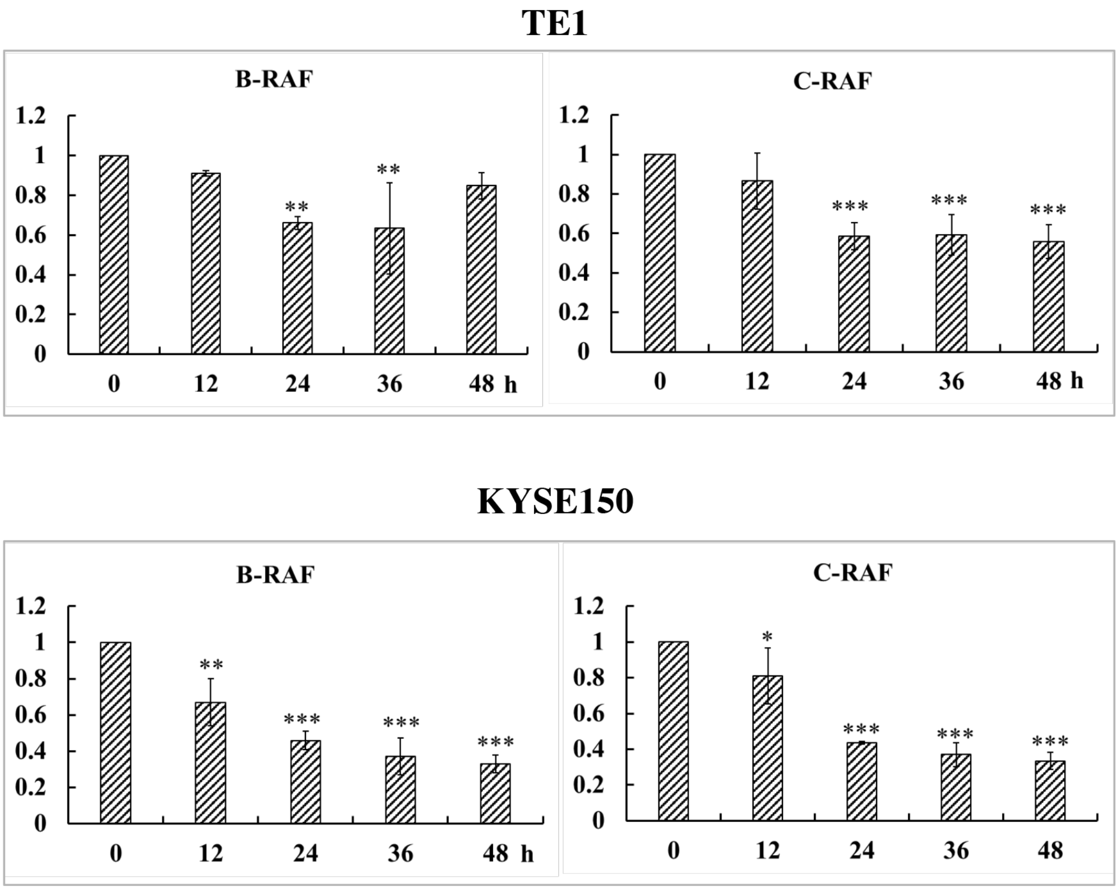

Supplementary Figure S6: GPX inhibits B-RAF and C-RAF mRNA levels. After treated with 20 μM GPX, RNA was extracted and the B-RAF and C-RAF mRNA levels were analyzed by Real-Time PCR. Data are analyzed by One-way ANNOVA and presented as the means ± S.D. *$P < 0.05$, **$P < 0.01$, ***$P < 0.001$.

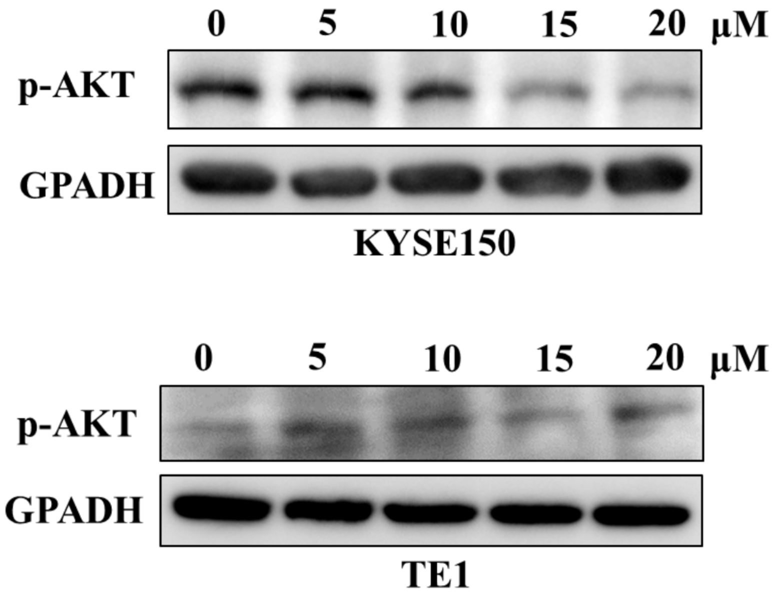

Supplementary Figure S7: GPX suppresses AKT phosphorylation level. After treated with 20 μM GPX, cells were lysed, and AKT phosphorylation was examined by western blot.

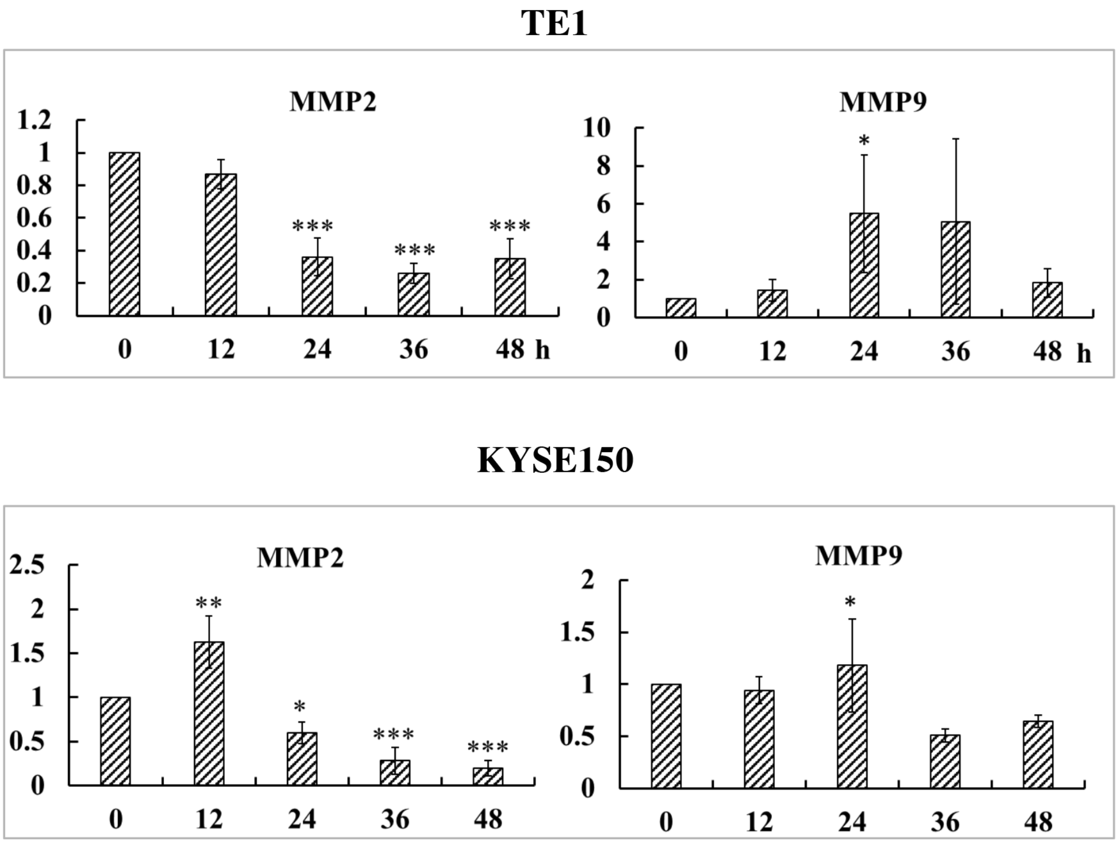

Supplementary Figure S8: GPX suppresses MMP-2 mRNA levels. TE1 and KYSE150 cells were treated with GPX, and RNA was extracted and reverse transcribed. MMP-2 and MMP-9 mRNA levels were analyzed by Real-Time PCR System. Data are analyzed by One-way ANNOVA and presented as the means ± S.D. *$P < 0.05$, **$P < 0.01$, ***$P < 0.001$.

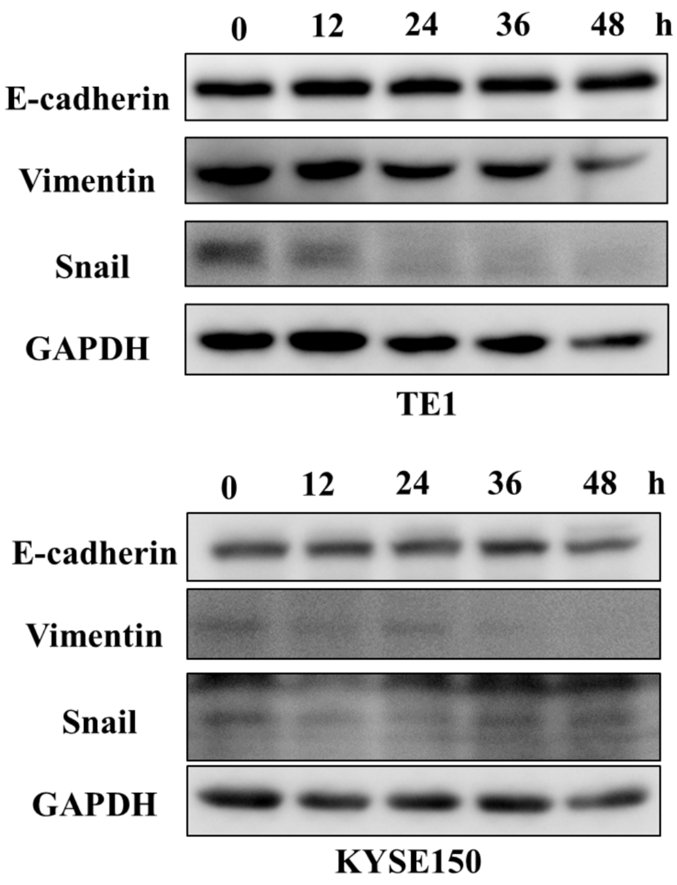

Supplementary Figure S9: GPX suppresses epithelial-mesenchymal transition. Three EMT markers: E-cadherin, Vimentin, Snail were analyzed by western blot in TE1 cells after treatment with 20 μM GPX at different time points.

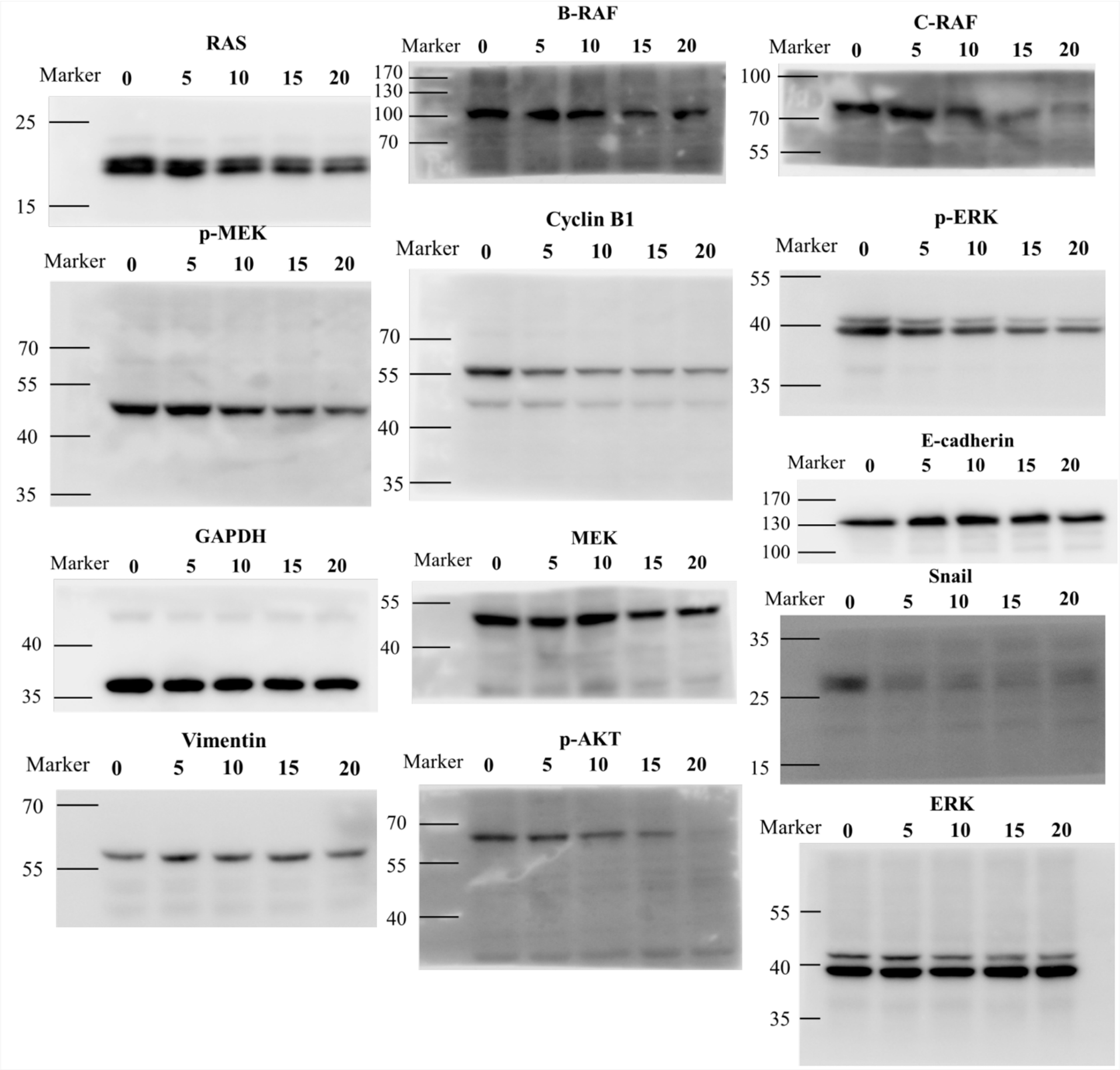

Supplementary Figure S10: Full-length blots of RAS, B-RAF, C-RAF, MEK, p-MEK, ERK, p-ERK, GAPDH, CyclinB1, p-AKT, E-cadherin, Vimentin and Snail proteins were presented when cancer cells were treated with different concentrations of GPX. One representative image of each protein is showed and experiments were performed at least three times.

Supplementary Table S1: Compounds for RAF inhibitors screening

No	Name
1	Neogambogic acid
2	Neobractatin
3	Isobractatin
4	Oblongifolin M
5	Oblongifolin L
6	1,5-Dimethyl Citrate
7	Morelloflavone
8	Griffipavixanthone
9	Cambogin
10	1,3,6,7-Tetrahydroxyxanthone
11	Guttiferone F
12	Oblongifolin C
13	Guttiferone K
14	Nujiangexanthone A
15	5,5′,7,7′-tetrahydroxy-2,2′-bis(4-hydroxyphenyl)-[3,8′-bichroman]-4,4′-dione
16	Volkensiflavone
